# A genomic overview of short genetic variations in a basal chordate, *Ciona intestinalis*

**DOI:** 10.1186/1471-2164-13-208

**Published:** 2012-05-30

**Authors:** Yutaka Satou, Tadasu Shin-i, Yuji Kohara, Nori Satoh, Shota Chiba

**Affiliations:** 1 Department of Zoology, Graduate School of Science, Kyoto University, Sakyo Kyoto 606-8502, Japan; 2 National Institute of Genetics, Mishima, Shizuoka, 411-8540, Japan; 3 Marine Genomics Unit, Okinawa Institute of Science and Technology, Onna, Okinawa 904-0495, Japan

**Keywords:** *Ciona intestinalis*, Short genetic variations, Single nucleotide polymorphisms (SNPs), Ascidian

## Abstract

**Background:**

Although the *Ciona intestinalis* genome contains many allelic polymorphisms, there is only limited data analyzed systematically. Establishing a dense map of genetic variations in *C. intestinalis* is necessary not only for linkage analysis, but also for other experimental biology including molecular developmental and evolutionary studies, because animals from natural populations are typically used for experiments.

**Results:**

Here, we identified over three million candidate short genomic variations within a 110 Mb euchromatin region among five *C. intestinalis* individuals. The average nucleotide diversity was approximately 1.1%. Genetic variations were found at a similar density in intergenic and gene regions. Non-synonymous and nonsense nucleotide substitutions were found in 12,493 and 1,214 genes accounting for 81.9% and 8.0% of the entire gene set, respectively, and over 60% of genes in the single animal encode non-identical proteins between maternal and paternal alleles.

**Conclusions:**

Our results provide a framework for studying evolution of the animal genome, as well as a useful resource for a wide range of *C. intestinalis* researchers.

## Background

*Ciona intestinalis* is a chordate with a simpler and more compact genome than found in vertebrates or even cephalochordates, making it a useful model for studying chordate evolution. In 2002, we decoded the genome of a single *C. intestinalis* animal from a natural population (Half Moon Bay, California, USA) [1]. From the study, the 160 Mb *C. intestinalis* genome was shown to contain 110 Mb of euchromatin regions that encode approximately 16,000 genes, yielding an average gene density of one gene per 6.8 kb of DNA.

Based on the frequency of allelic polymorphisms of this single animal, the difference between two haplotypes was estimated at 1.2%. In vertebrates, millions of single nucleotide polymorphisms (SNPs) and insertions/deletions (indels) have also been reported. For example, genome sequencing of multiple human individuals has identified approximately three million SNPs per individual, which account for 0.1% of the haploid genome size [2-4]. In zebrafish and fugu, allelic polymorphisms within a single individual comprise 0.4% of the genome [5,6]. However, higher heterozygosity within individuals has been reported in several invertebrate deuterostomes, including sea urchin (4−5%) [7], amphioxus (4.0%) [8] and two other tunicates (4.5% in *Ciona savignyi* and 2.2% in *Oikopleura dioica*) [9-13].

*C. intestinalis* has been used for a wide range of biological studies including developmental and evolutionary studies. Establishing a dense map of genetic variations in *C. intestinalis* is essential for these studies, because no inbred strains are available and animals from natural populations are used for experiments. Genetic variation might affect reproduction of results obtained from different animals, and thus the map would be useful for designing and interpreting a wide range of molecular biology experiments. At the same time, genetic variation might be useful for annotating the genome, because functional elements including tissue-specific enhancers are thought to include less variation [14].

In most genome projects, DNA from only one individual was sequenced, and systematic genome-wide analyses of multiple individuals were rarely performed. As part of the *C. intestinalis* genome project, we also sequenced the genomes of three individuals collected from a natural population in Onagawa, Miyagi, Japan [1]. However, these reads were not incorporated into the final assembly due to the high degree of polymorphism. In the present study, we re-map all of the whole-genome shotgun reads generated in that project and identify short genetic variations to better understand the nature of high polymorphic genomes. This information will also be useful for experimental biologists, who typically use animals whose genetic backgrounds differ from the reference sequence.

## Results and discussion

### Identification of short genetic variations

In the present study, we used only high quality regions (quality value ≥ 25) of raw shotgun reads from three different sets of genomic DNAs, which we used for determining the draft genome sequence of *C. intestinalis*[1]. All of the sequence data were generated by the Sanger method [15]. The first dataset (US1) is from a single individual from Half Moon Bay, California, USA, and gave rise to most of the sequences incorporated into the draft assembly. The second small set (US2) is from a different individual from the same population and also was included in the assembly. The third set (JP) is from a mixture of three individuals from a natural population in Japan. Because a preliminary inspection of this third dataset revealed substantial differences from the first and second sets, these sequences were not incorporated into the first draft assembly [1].

As shown in Table 1, we retrieved 2,815,455, 60,882, and 2,318,633 sequence fragments from the US1, US2 and JP datasets, respectively, with 2,240,089 (79.6%), 43,032 (70.7%) and 1,711,246 (73.8%) successfully mapped onto the reference genome under a stringent condition. We used the most recent assembly (KH assembly) as the reference, which represents a haploid genome based on the US1 and US2 datasets. Even though 20−30% of sequences were not mapped, the current genome assembly is thought to cover most of the euchromatic regions, which comprise 70% of the entire genome [1,16]. The average depth of coverage was 5.4-, 0.09-, and 6.7-fold (Additional file 1 Figure S1). 97.5% and 94.8% of the genome sequence was covered by at least one US1 and one JP read, respectively (99.2% in total). Similarly, 92.1% and 89.0% of the genome was covered by two or more US1 and JP reads. The US2 dataset covered 8,514,691 nucleotides (7.6%), mostly by single reads.

**Table 1:** Sequences used in the present study

	**US1 dataset**	**US2 dataset**	**JP dataset**
No. sequence fragments	2,815,455	60,882	2,318,633
No. sequence fragments mapped	2,240,089	43,032	1,711,246

From the alignments, we identified 3,233,449 SNPs and 618,609 indels (284,803 insertions and 333,806 deletions) (Table 2). Indels ranged from 1 to 75 base pairs (note that the method used in the present study cannot identify long indels). Because the total length of all contigs in the current assembly (KH assembly) is 112 Mb [16], the candidate genetic variations found in the present study correspond to 3.4% of the genome.

**Table 2:** Summary of candidate short genetic variations found in the present study

	**SNPs**	**indels**
Candidate sites Total	3,233,449	618,609
Sites represented by only one read	1,095,119	240,873
Sites represented by multiple reads	2,138,330	377,736

Among all SNPs, 59% were A:T↔G:C transitions. Among the remaining 41%, C:G↔G:C transversions (6%) were represented much less than less than A:T↔C:G (19%) and A:T↔T:A (16%) transversions.

Among the candidates, 2,516,066 were represented by multiple reads, and they were considered to represent true genetic variations with high probability. The remaining 1,335,992 (1,095,119 SNPs and 240,873 indels) were represented by only one read, and we estimated the error rate of them and the minimum number of true variations. Based on quality values, 198,777 sequence errors are expected in the entire datasets. Even if all of these errors are included among these 1,334,992 candidates, a large fraction (~85%) is expected to represent true genetic variations.

To estimate the minimum number of true variations represented by single reads, we compared them with 1,179,850 of ESTs. Of these ESTs, 73% are derived from animals sampled from Onagawa, Miyagi, Japan, where we sampled the animals for generating the JP dataset. Among 1,095,119 substitutions, 203,286 sites were covered by one or more ESTs, and in 78,862 positions (39%), the same variations were observed in ESTs. This suggests that at least 39% of the candidate variations represented by only one read are true variations. Note that this latter estimation is very conservative and may be much lower than the real; more variations could be found in common if we had more ESTs. If this error estimation (roughly 39–85%) can be applied to indels as well, genetic variations may account for 2.7–3.3% of nucleotide positions among the five animals.

Since our method cannot identify long indels and chromosomal rearrangements, this may be an underestimate of the genetic differences between the animals. On the other hand, copy number variations might lead to mis-identification of genetic variations. Nonetheless, the genetic variations we identified provide an overview of short genetic variations in *C. intestinalis*.

### Experimental validation of short genetic variations

To validate the identified genetic variations, we amplified five short genomic regions on five different chromosomes from 11 different animals by PCR and determined their sequences. These animals were randomly chosen among offsprings of 70 animals sampled from the same place where we had took the animals for generating the JP dataset. Of the 148 SNPs and 20 indels originally identified within these five regions, 120 (71%) were confirmed in one or more animals examined (Additional file 1 Table S1 and Additional file 1 Table S2). The remaining 29% may include bona fide variations that could be confirmed by sampling more individuals.

### Differences between and within the three datasets

**Figure 1:**
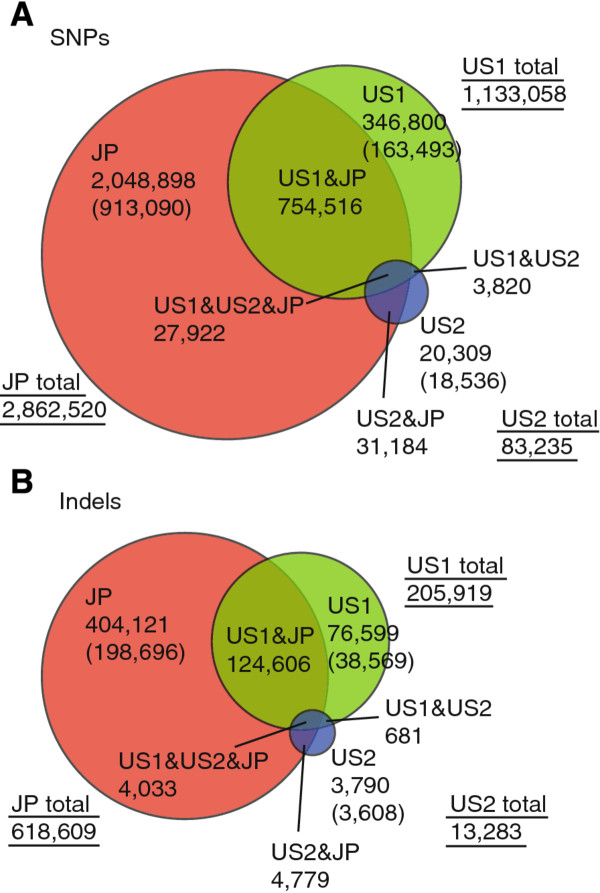
**Distribution and overlap of genetic variations identified in three datasets.** Venn diagrams show the distribution and overlap of **(A)** SNPs and **(B)** indels identified in three datasets. Circle size is proportional to the number of variations found in each of the datasets. Numbers in parentheses represent variations that were found in one read only.

The mapping of the US1 dataset showed 1,338,977 sites of differences, with 1,133,058 being SNPs and 205,919 being indels (Figure 1; 1.19% of the entire genome sequence). Among these, 202,062 variations (163,493 SNPs and 38,569 indels) were represented by only one read. If we estimate that 39-85% are real, 78,804-171,753 (39-85% of 202,062) should be true variants. Therefore, together with the 1,136,915 sites represented by multiple reads, 1,215,719-1,308,668 are estimated to represent true genetic variations in total (1,033,327-1,108,534 SNPs and 182,392-200,134 indels). This corresponds to a 1.08-1.17% difference between alleles of the reference individual, slightly lower than a previous rough estimate of 1.2%.

A similar calculation indicated that six haploid genomes from three individuals of a single population in Japan included 2,721,870-3,233,291 (2.43-2.88% of the entire genome) sites that differed from the reference genome. Of these, 2,288,273 sites were represented by multiple JP reads. At 357,229 sites, all of the JP reads differed from the reference. Because these 357,229 sites were not polymorphic among the JP reads, it is possible that these sites are homozygous in the three Japanese individuals, and represent genetic variations between the two populations. The average number of pairwise differences between the Japanese individuals in regions covered by two or more JP reads covered (99.8Mb) was 1,089,997 and the average number of pairwise differences per site (nucleotide diversity) was 0.0109. These values were close to the SNP sites and frequencies observed in the US1 set. Thus, the nucleotide diversity of *C. intestinalis* is lower than those of *C. savignyi*, sea urchin and amphioxus but much higher than those of humans [2-4] and fish [5,6].

Among 3,233,449 SNPs and 618,609 indels, 813,622 (25.2%) and 133,418 (21.6%) were found in both the JP and the US (US1 and US2) datasets (Figure 1). This suggests that a significant fraction of genetic variations are shared between the two populations and are not minor alleles. If we obtain more sequence data from different individuals in future, more variations would turn out to be shared between the two populations. At the same time, these data also suggest that *C. intestinalis* possesses a large gene pool, because we found genetic variations taking ~3.3% of the genome from only the five animals of the two populations. If we obtain more sequence data from different individuals in future, more novel variations would be found. Indeed, in the above validation experiment, we identified 30 additional variations over 3.6 kb (data not shown), suggesting there are still unknown genetic variations in the gene pool of this animal.

### Characterization of the short genetic variations

**Figure 2:**
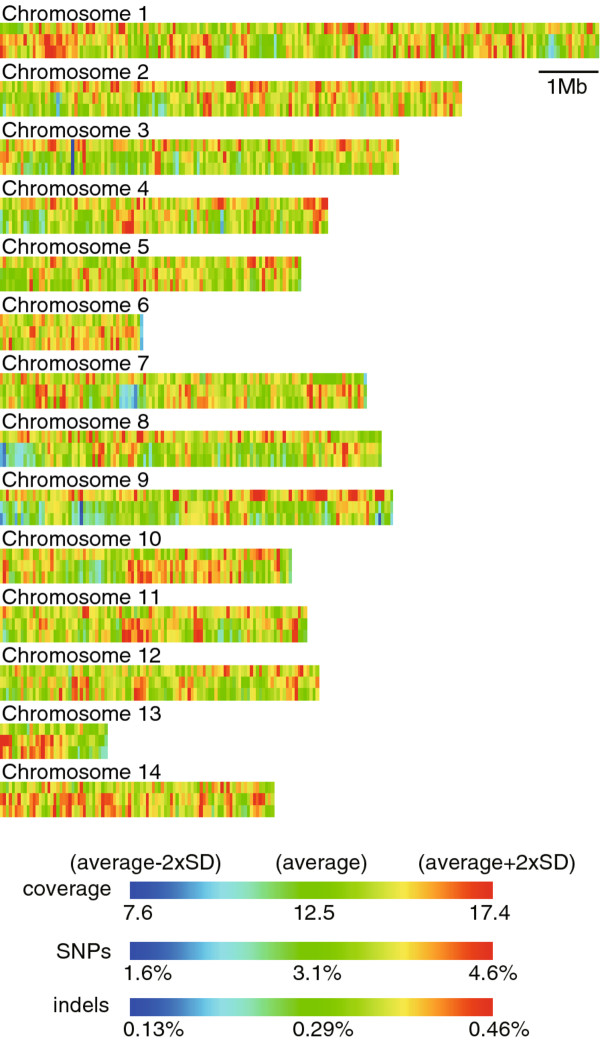
**SNPs and indels are not evenly distributed over the chromosomes.** The map of sequence coverage (top bar of each chromosome) and density of SNPs (middle bar) and indels (bottom bar) across the 14 chromosomes were constructed in non-overlapping 50 kb windows, which are represented by colored rectangles. The color scales are between 2xSDs of either side of the averages. The averages and SDs were calculated within the chromosomes, in which 68% of the assembly is included.

Figure 2 shows the sequence coverage and density of SNPs and indels in non-overlapping 50 kb chromosomal windows. Genetic variations identified in the present study were not necessarily evenly distributed over the genome. Therefore, we next examined the variations at higher resolution. Hereafter, we describe nucleotide substitutions and indels represented by two or more reads, unless otherwise noted.

**Figure 3:**
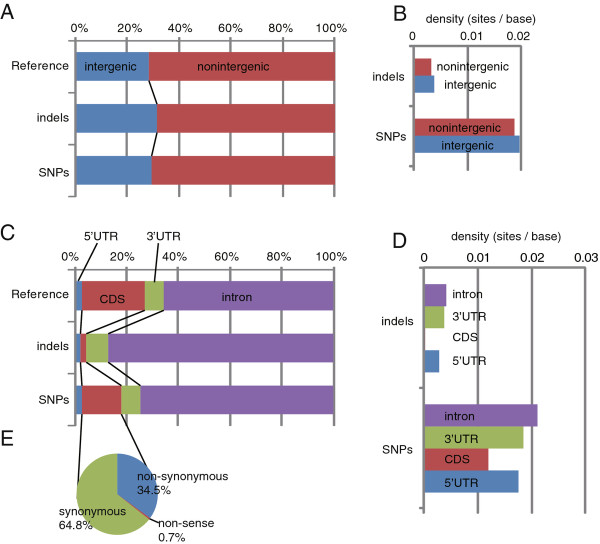
**Identified SNPs and small indels.(A)** Ratio of intergenic and non-intergenic (gene) regions across the entire genome and among SNPs and indel sites. **(B)** Density of SNPs and indels in intergenic and non-intergenic regions. **(C)** Ratio of length of 5’-UTRs, coding sequences, 3’-UTRs and introns within gene regions across the entire genome and among SNPs and indel sites. **(D)** Density of SNPs and indels in 5’-UTRs, coding sequences, 3’-UTRs and introns. **(E)** Ratio of non-synonymous/nonsense and synonymous SNPs found in coding sequences.

In *C. intestinalis*, intergenic regions comprise 28.7% of the genome. Among the identified variations, 29.6% of the SNPs and 31.5% of the indels were found in intergenic regions (Figure 3A). The density of SNPs in the intergenic region was 0.020 (1 per 50 bp), while that in gene regions was 0.019 (Figure 3B). Among the variations mapped to gene regions, 74.4% of SNPs were found in introns, while the current gene models (KH models) predict that introns comprise 65.7% of gene regions (Figure 3C). Similarly, 2.5%, 7.4% and 15.6% of SNPs were found in 5’- and 3’-UTRs and coding sequences, while 5’- and 3’-UTRs and coding sequences account for 2.6%, 7.4% and 24.3%. Namely, the density of SNPs was highest in introns (0.021) and lowest in coding regions (0.012; Figure 3D). The density of SNPs in the intergenic regions was slightly lower than but almost comparable to that in introns.

Among the SNPs found in coding sequences, 34.5% caused non-synonymous amino acid changes in encoded proteins, 0.7% caused nonsense mutations and the remaining 64.8% did not change the amino acid sequence (Figure 3E). On average, we found 5.6 non-synonymous, 0.1 nonsense and 10.4 synonymous SNPs per gene. In the reference genome, 15,254 gene models are predicted [16]. The present study identified SNPs that caused non-synonymous and nonsense alternations in 12,493 (81.9%) and 1,214 (8.0%) genes, respectively. Even when the US1 dataset is examined, 9,347 (61.3%) and 610 (4.0%) genes showed non-synonymous and nonsense changes, respectively. On the other hand, 87.52% indels were found in introns, and 2.3%, 8.8% and 1.9% were found in 5’-UTRs, 3’-UTRs and coding regions (Figure 3C). Thus the density of indels was much lower in coding regions than in introns, 5’-UTRs and 3’-UTRs (Figure 3D).

### Genetic variations and gene function

From 11,700 high-quality gene models with clear ORFs, we calculated the ratio of the rate of non-synonymous substitutions (dN) to that of synonymous substitutions (dS) to be 0.16. In contrast, the closely related, *Ciona savignyi* genome shows a much lower dN/dS ratio (0.07) [13]. The dN/dS ratio in *C. intestinalis* was more similar to that of zebrafish (dN/dS =0.14) [6], suggesting relaxed selection pressure compared with *C. savignyi*.

For functional annotations, we first compared the whole *C. intestinalis* proteome with the human proteome using the Blastp program [17], because the human proteome is the most thoroughly curated and annotated and because both of *C. intestinalis* and humans belong to the same phylum, Chordata. Then, gene ontology (GO) terms from the best-hit human protein for each *C. intestinalis* protein were used in the following analysis.

**Figure 4:**
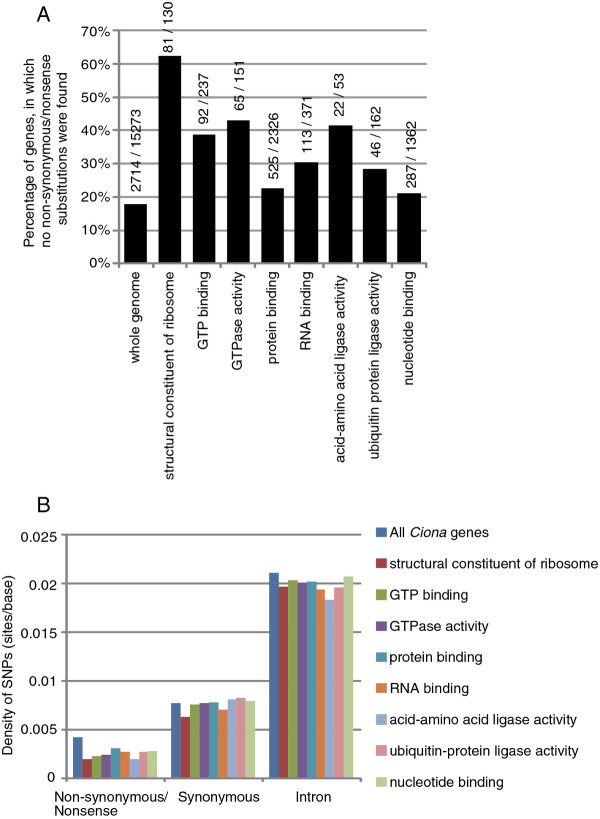
**Function of genes with no non-synonymous and nonsense substitutions.(A)** Functional GO categories overrepresented among genes with no non-synonymous/nonsense substitutions. The numbers above the bars indicate the number of genes with no non-synonymous/nonsense substitutions, and the number of total genes in each of the functional groups. **(B)** Density of non-synonymous/nonsense, synonymous and intronic SNPs within each of the functional groups.

We found a small number of GO terms were significantly overrepresented in genes with no non-synonymous/nonsense substitutions. Under the molecular function ontology, GO terms of structural constituent of ribosome, GTP binding, GTPase activity, protein binding, RNA binding, acid-amino acid ligase activity, ubiquitin-protein ligase activity and nucleotide binding were overrepresented (Figure 4A; z-test, p < 0.01). Because the most extremely overrepresented term was structural constituents of ribosomes, we manually inspected all of the genes encoding ribosomal proteins. No non-synonymous/nonsense substitutions were found in 69 of 78 genes encoding non-mitochondrial ribosomal proteins and in 14 of 49 mitochondrial ribosomal proteins. Most of the remaining genes encoding ribosomal proteins also contained fewer non-synonymous/nonsense substitutions (Additional file 1 Table S3).

The average length of proteins associated with these overrepresented GO terms was shorter than that of proteins in the entire genomes (236 and 447 amino acids, respectively). However, this difference is not directly relevant; the density of non-synonymous/nonsense substitutions were markedly lower in genes encoding proteins associated with these GO terms than in the entire genome, while synonymous and intronic SNPs were found at almost the same frequency or slightly less frequently (Figure 4B). Thus, genes encoding proteins associated with these overrepresented GO terms have less non-synonymous/nonsense substitutions.

We calculated dN/dS ratios of the *C. intestinalis* genes encoding proteins with human homologs (8,263 genes). Among them, 126 genes showed a dN/dS larger than 1, and therefore they are candidate genes under positive selection pressure. However, we could not identify a shared biological function among them.

### Short genetic variation data as a resource for experimental biologists

Because no *C. intestinalis* inbred or laboratory strains have been established and researchers are using animals from natural populations, the genetic backgrounds of these animals differ from each other and from the reference. Therefore, we integrated the short genetic variations identified in the present study into the ghost database, a major *Ciona* database with a large set of genome-wide data [16,18], while all of the sequence data have been available in the public database [1]. Users can browse these variations as a track of the genome browser, each of which is linked to a detailed description. The information will be helpful not only for linkage analysis but also for designing and interpreting a wide range of molecular biology experiments.

## Conclusions

When we had published the first genome sequence of *C. intestinalis* in 2002 [1], we were aware that this genome was extremely polymorphic. However, our present data indicate that the *C. intestinalis* genome is less polymorphic than the genomes of other invertebrate species that have been sampled from natural, non-inbred populations. This may be related to the fact that among these species, *C. intestinalis* has the smallest and most compact genome, with the exception of *Oikopleura*, which has a larger population size and an extraordinary short generation time [10]. In addition, the efficiency of proofreading system during genome replication and the genome repair systems might be different among these species.

Our results suggest that *C. intestinalis* has a huge gene pool, and that researchers using these animals must be aware of the possible effects of genetic variation in their experiments. Indeed, genetic variation might affect reproduction of results obtained from different laboratories, or even from different animals.

At the same time, our results will be useful for annotating the genome, because a previous study showed that intraspecies sequence comparisons could effectively identify candidates of tissue-specific enhancers [14].

## Materials and methods

### Sequence reads and alignments

The raw shotgun sequence reads were generated in the previous study [1], which can be found in the trace archive of the public database. From these sequences, we extracted regions with a stretch of 100 or more bases, each of which has a high-quality value (≥ 25).

These high-quality sequences were aligned against the reference genome (KH assembly) [16] using the ssaha2 program [19]; the mapping criterion was identity of 90% or more of the entire read length. From the alignments, we identified short genetic variations (Additional file 2 Table S4, Additional file 3 Table S5, Additional file 4 Table S6). The KH gene models were used for identifying gene regions [16]. When multiple transcripts were predicted for a single gene locus, we used transcripts with the longest coding sequences. We estimated dN/dS by using CODEML [20] with the F3 x 4 codon frequency model from concatenated coding sequences of 11,700 genes, which have clear ORFs beginning with ATG and ending with a stop codon.

### Experimental validation of short genetic variations

*Ciona intestinalis* adults were cultivated at the Maizuru Fisheries Research Station of Kyoto University, in Maizuru, which faces the Sea of Japan. These animals are offsprings of 70 animals that were sampled from Onagawa, Miyagi, Japan. Genomic DNA was isolated from the sperm of 11 different individuals.

We amplified genomic fragments by polymerase-chain-reactions with five different sets of primers. PCR primers used are shown in Additional file 1 Table S7. The amplified sequence fragments were directly sequenced using an ABI3130xl sequencer.

### Gene ontology analysis

All of the predicted *C. intestinalis* proteins were subjected to a BLAST search against the human proteome using uniprotKB (threshold value < 1e-5) [21]. The best hit human proteins were chosen as likely orthologs, and GO annotations for these candidate orthologs were used.

## Abbreviations

SNP, single nucleotide polymorphisms; Indels, insertions/deletions; EST, expressed sequence tag.

## Competing interests

The authors declare that they have no competing interests.

## Authors' contributions

YS designed the study and analyzed the data. SC validated polymorphisms experimentally. TS, YK and NS contributed to generating whole-genome shotgun data from the JP dataset and the initial stage of data mining. YS wrote the paper. All authors read and approved the final manuscript.

## Supplementary Material

Additional file 1 **Figure S1.** Distribution of sequence coverage of the reference genome, **Table S1.** Genetic variations in five genomic regions of 11 different animals tested in the present study, **Table S2.** Numbers of genetic variations found in 11 different animals and in whole genome shotgun reads, **Table S3.** Numbers of synonymous and non-synonymous SNPs in genes encoding ribosomal proteins, **Table S7.** Primers used in the present study. Click here for file

Additional file 2 **Table S4.** SNPs identified in the present study. Click here for file

Additional file 3 **Table S5.** Insertions identified in the present study. Click here for file

Additional file 4 **Table S6.** Deletions identified in the present study. Click here for file
