# Supplementary material for: A genomic overview of short genetic variations in a basal chordate, Ciona intestinalis
Source: BMC Genomics. 2012 May 30;13:208. doi: 10.1186/1471-2164-13-208 (PMC3424144; doi:10.1186/1471-2164-13-208)
Supplement: Additional file 1 — Figure S1. Distribution of sequence coverage of the reference genome, Table S1. Genetic variations in five genomic regions of 11 different animals tested in the present study, Table S2. Numbers of genetic variations found in 11 different animals and in whole genome shotgun reads, Table S3. Numbers of synonymous and non-synonymous SNPs in genes encoding ribosomal proteins, Table S7. Primers used in the present study. [file 1471-2164-13-208-S1.pdf]

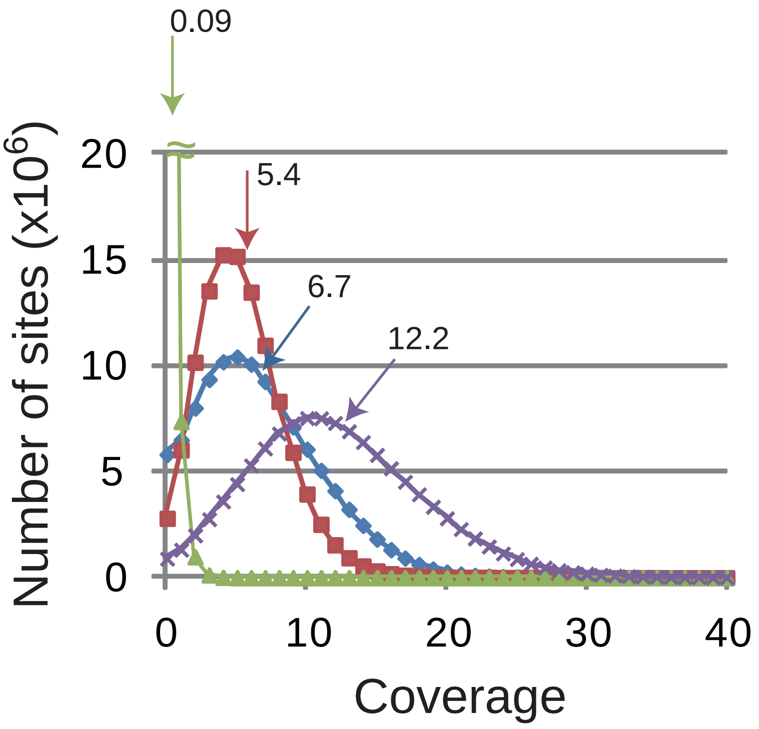

Figure S1. Distribution of sequence coverage of the reference genome. Red, light green and blue lines indicate distribution of sequence coverage by the US1, US2 and JP datasets, respectively. The purple line indicates distribution of total sequence coverage of the three datasets. Averages are indicated by arrows.

Table S1. Genetic variations in five genomic regions of 11 different animals

Chromosome	Nucleotide position	Nucleotide in the reference	Variarions identified	Individuals 1	2	3	4	5	6	7	8	9	10	11
1	16308	C	T	.	.	.	.	.	.	C/T	.	.	.	C/T
1	16357	A	G	A/G	G	G	.	G	A/G	A/G	A/G	.	A/G	.
1	16367	A	G	A/G	G	G	.	G	A/G	A/G	A/G	.	A/G	.
1	16374	C	T	C/T	C/T	C/T	.	.	.	C/T	C/T	.	C/T	.
1	16392	A	T	A/T	T	T	T	T	A/T	T	T	A/T	T	A/T
1	16400	G	A	.	.	.	.	.	.	.	.	.	.	.
1	16415	T	C	T/C	C	C	C	C	T/C	C	C	T/C	C	T/C
1	16435	G	A	G/A	A	A	A	A	G/A	A	A	G/A	A	G/A
1	16444	C	A	A/C	A	A	A/C	A	C/A	C/A	A	.	A	.
1	16447	C	G	C/G	G	G	C/G	G	C/G	C/G	G	.	G	.
1	16450	T	Deletion	hetero	homo	homo	hetero	.	hetero	hetero	homo	.	homo	.
1	16461	C	T	-	.	.	-	.	-	-	.	.	.	.
1	16472	G	A	-	G/A	G/A	-	.	-	-	G/A	.	G/A	.
1	16482	C	T	-	C/T	C/T	-	.	-	-	C/T	.	C/T	.
1	16497 *	T	C	-	.	.	-	.	-	-	T/C	.	.	.
1	16499	A	G	-	.	.	-	.	-	-	.	.	.	.
1	16509	A	T	-	T	T	-	.	-	-	T	T	T	T
1	16541	A	G	-	G	G	-	G	A/G	-	G	A/G	G	A/G
1	16542	C	A	-	A	A	-	A	C/A	-	A	C/A	A	C/A
1	16545	A	C	-	C	C	.	C	.	-	.	.	.	.
1	16575	G	T	-	G/T	G/T	.	.	.	-	G/T	.	G/T	.
1	16579		Insertion	hetero	hetero	hetero	.	.	.	hetero	hetero	.	hetero	.
1	16582 *	C	A	.	.	.	C/A	.	.	.	.	C/A	.	.
1	16594 *	T	A	.	.	.	T/A	.	.	.	.	T/A	.	.
1	16634	C	T	C/T	C/T	C/T	.	.	.	C/T	C/T	.	C/T	.
1	16678	G	A	G/A	A	A	G/A	A	G/A	A	A	.	A	G/A
1	16679	T	G	T/G	G	G	T/G	G	T/G	G	G	.	G	T/G
1	16695	T	C	.	.	.	.	.	T	T/C	.	.	.	T/C
1	16701 *	G	A	.	.	.	.	.	.	G/A	.	.	.	G/A
2	158669	T	C	C	C	T/C	.	C	C	T/C	.	.	T/C	T/C
2	158734	G	A	A	A	G/A	A	G/A	A	G/A	A	G/A	A	A
2	158755	G	C	.	.	.	.	.	.	.	.	.	.	.
2	158771 *	G	C	.	.	.	.	.	.	.	.	.	.	.
2	158787 *	C	T	.	.	.	T	.	.	.	T	C/T	C/T	C/T
2	158804	TT	Deletion	homo	hetero	.	.	homo	hetero	.	.	.	.	hetero
2	158808	ACACTTT	Deletion	homo	-	.	.	hetero	-	.	.	.	.	-
2	158838	A	T	.	-	.	.	-	-	.	.	A/T	.	-
2	158870	T	A	.	-	.	.	-	-	.	.	.	.	-
2	158885	A	T	.	-	T/A	.	-	-	T/A	.	.	T/A	-
2	158891	A	T	T	-	.	.	-	-	.	.	.	.	-
2	158905	C	Deletion	.	-	hetero	homo	-	-	hetero	homo	-	homo	-
2	158944	C	A	A	-	-	A	-	-	-	A	C/A	A	-
2	158956	GTGTATCTTGATAC	Deletion	.	-	-	homo	-	-	-	homo	hetero	hetero	-
2	158980	A	T	.	-	-	T	-	-	-	T	-	-	-
2	158982	A	G	.	-	-	G	-	-	-	G	-	-	-
2	159021	A	G	.	-	-	.	-	-	-	.	-	-	-
2	159039	G	A	.	-	-	.	.	-	-	.	-	-	-
2	159060	A	G	.	-	-	G	.	-	-	G	-	-	-
2	159072	A	G	.	-	-	.	.	-	-	.	-	-	-
2	159077	A	G	.	-	-	G	.	-	-	G	-	-	-
2	159087	T	Deletion	.	-	-	.	.	-	-	.	-	-	-
2	159097	T	C	C	-	-	.	T/C	-	-	.	-	-	-
2	159147	T	A	.	-	-	A	.	-	-	A	-	-	-
2	159148	A	T	.	-	-	.	.	-	-	.	-	-	-
2	159149	T	A	A	-	-	A	T/A	-	-	A	-	-	-
2	159195	AATA	Deletion	.	-	-	.	.	-	-	.	-	-	-
2	159261	T	C	.	-	-	C	.	-	-	C	-	-	-
2	159328	G	A	.	-	-	.	.	-	-	.	-	-	-
2	159337 or 159338	A	Deletion	.	-	-	homo	.	-	-	homo	hetero	-	hetero
2	159338 *	A	T	.	-	-	.	.	-	-	.	A/T	-	A/T
2	159340	G	C	.	-	-	.	.	-	-	.	.	-	.
2	159364	A	G	.	-	-	.	.	-	-	.	.	-	.
2	159369	G	A	.	-	-	.	.	-	-	.	.	-	.
2	159373	A	G	.	-	-	.	.	-	-	.	.	-	.
2	159415	A	T	.	-	-	T	.	-	-	T	A/T	-	A/T
2	159416 *	A	G	.	-	-	.	.	-	-	.	.	-	.
2	159419	G	C	C	-	-	.	G/C	-	-	.	.	-	G/A
2	159426 *	T	A	.	-	-	.	.	-	-	.	.	-	.
2	159450	A	G	.	-	-	.	.	-	-	.	.	-	.
2	159451	G	T	.	-	-	.	.	-	-	.	.	-	.

2	159458		C	G	.	-	-	.	.	-	-	.	.	-	.
2	159498		T	A	.	-	-	.	.	-	-	.	.	-	.
2	159504	*	G	A	.	-	-	.	.	-	-	.	.	-	.
2	159513		T	C	.	-	-	.	.	-	-	.	.	-	.
2	159557		G	A	A	G/A	.	.	G/A	G/A	.	.	.	.	G/A
2	159569		A	C	.	.	.	.	.	.	.	.	.	.	.
2	159582		T	C	.	T/C	T/C	.	.	T/C	T/C	.	T/C	T/C	T/C
2	159602		A	C	.	A/C	A/C	.	.	A/C	A/C	.	A/C	A/C	A/C
2	159625		A	G	.	.	.	.	.	.	.	.	.	.	.
2	159665		C	T	-	-	-	-	-	-	-	-	-	T	T
3	3549893		G	A	A	A	A	A	A	.	G/A	A	A	A	A
3	3549948	*	G	A	.	.	.	.	.	G/A	.	.	.	.	.
3	3550041		G	A	A	A/G	A/G	A/G	A	.	G/A	G/A	A	.	A
3	3550047		A	T	T	A/T	A/T	A/T	T	.	A/T	A/T	T	.	T
3	3550058	*	C	T	T	.	T	T	.	.	T	.	T	.	.
3	3550059	*		Insertion	hetero	.	hetero	hetero	.	.	hetero	.	homo	.	.
3	3550081		C	T	C/T	C/T	-	-	.	C/T	-	-	-	T	.
3	3550115		G	A	G/A	G/A	-	-	A	.	-	G/A	.	.	A
3	3550128		G	A	G/A	.	-	-	.	G/A	-	.	A	.	.
3	3550130		G	C	G/C	G/C	-	-	C	.	-	G/C	.	G/C	C
3	3550134		T	C	T/C	T/C	-	-	.	C	-	T/C	C	C	.
3	3550136		A	T	.	.	-	-	.	A/T	-	.	.	.	.
3	3550137		T	G	.	.	-	-	.	T/G	-	.	.	.	.
3	3550158		C	T	.	.	-	-	.	C/T	-	.	.	.	.
3	3550180		G	A	A	A	-	-	A	A	-	A	A	A	A
3	3550219		G	T	T	T	-	-	T	T	-	T	T	T	T
3	3550222		A	T	.	.	-	-	.	-	-	.	.	.	.
4	3241059		G	A	-	-	-	-	-	-	-	.	-	.	.
4	3241100		T	G	.	.	.	.	T/G	.	.	.	.	.	.
4	3241150		A	G	.	A/G	A/G	.	.	A/G	.	A/G	.	.	A/G
4	3241189		A	G	.	A/G	.	A/G	A/G	.	.	.	.	.	A/G
4	3241201		T	C	.	.	.	.	T/C	.	.	.	.	.	.
4	3241204		A	T	.	A/T	.	A/T	.	.	.	.	.	T	A/T
4	3241206		T	A	.	T/A	.	T/A	T/A	.	.	.	.	.	T/A
4	3241216		T	A	.	T/A	A	T/A	T/A	.	.	.	.	A	T/A
4	3241223		G	C	.	.	.	.	G/C	.	.	.	.	.	.
4	3241243		C	T	.	C/T	.	C/T	.	.	.	.	.	T	.
4	3241252		G	A	.	G/A	.	G/A	.	.	.	.	.	A	G/A
4	3241253		T	C	.	T/C	T/C	.	.	T/C	.	T/C	.	.	T/C
4	3241258		C	T	.	C/T	.	C/T	C/T	.	.	.	.	T	.
4	3241268	*	A	T	.	.	.	A/T	-	.	.	A/T	.	.	.
4	3241271		T	G	.	T/G	.	T/G	-	.	.	.	.	G	T/G
4	3241292		A	G	.	A/G	A/G	.	-	A/G	.	A/G	.	.	A/G
4	3241297	*		Insertion	.	.	.	hetero	-	.	.	hetero	.	.	.
5	198495		C	T	.	C/T	.	-	.	.	.	-	-	.	.
5	198503		T	G	G	.	T/G	-	.	G	G	-	-	G	.
5	198514			Insertion	.	.	.	-	.	.	.	-	-	.	.
5	198535		C	Deletion	homo	hetero	homo	-	homo	homo	homo	-	-	homo	homo
5	198537		T	C	C	-	C	-	C	C	C	-	-	C	C
5	198548		T	Deletion	hetero	-	hetero	-	.	hetero	hetero	-	-	hetero	hetero
5	198553		G	A	-	-	-	-	.	-	-	-	-	-	-
5	198565		G	C	-	-	-	-	.	-	-	-	-	-	-
5	198605			Insertion	-	.	-	-	homo	-	-	-	-	-	-
5	198619		G	T	-	T	-	-	T	-	-	-	-	-	-
5	198646		A	G	-	.	-	-	.	-	-	-	-	-	-
5	198651		A	G	-	G	-	-	G	-	-	-	-	-	-
5	198657		T	C	-	C	-	-	C	-	-	-	-	-	-
5	198659		G	C	-	.	-	-	.	-	-	-	-	-	-
5	198690		C	T	-	.	-	-	.	-	-	-	-	-	-
5	198736		C	T	-	T	-	-	T	-	-	-	-	-	-
5	198744		T	C	-	C	-	-	.	-	-	-	-	-	-
5	198769		C	G	-	C/G	-	-	G	-	-	-	-	-	-
5	198787	*	A	Deletion	-	.	-	-	.	-	-	-	-	-	-
5	198794		T	Deletion	-	.	-	-	.	-	-	-	-	-	-
5	198821		G	T	-	G/T	-	-	T	-	-	-	-	-	-
5	198829		T	C	-	T/C	-	-	.	-	-	-	-	-	-
5	198834		G	T	-	.	-	-	.	-	-	-	-	-	-
5	198839		G	A	-	G/A	-	-	.	-	-	-	-	-	-
5	198872		C	T	-	.	-	-	.	-	-	-	-	-	-
5	198876		A	G	-	A/G	-	-	G	-	-	-	-	-	-
5	198878		T	C	-	.	-	-	.	-	-	-	-	-	-
5	198886		A	T	-	T	-	-	T	-	-	-	-	-	-
5	198936		G	Deletion	-	.	-	-	.	-	-	-	-	-	-
5	198966	*	T	C	-	.	-	-	.	-	-	.	.	-	-
5	198967		C	T	-	.	-	-	.	-	-	.	.	-	-
5	198972		C	T	-	.	-	-	.	-	-	C/T	C/T	.	.

5	199012	C	G	-	.	-	-	.	-	-	.	.	-	-
5	199014	A	G	-	.	-	-	.	-	-	A/G	A/G	-	-
5	199018 *	G	A	-	.	-	-	A	-	-	.	G/A	-	-
5	199042	G	A	-	.	-	-	.	-	-	.	.	-	-
5	199053	C	A	-	-	-	-	A	-	-	C/A	A	-	-
5	199073	T	G	-	-	-	-	G	-	-	T/G	G	-	-
5	199106	T	C	-	-	-	-	C	-	-	T/C	C	-	-
5	199117 *	A	T	-	-	-	-	T	-	-	A/T	T	-	-
5	199129 *	T	A	-	-	-	-	A	-	-	T/A	A	-	-
5	199146	A	T	-	-	-	-	.	-	-	.	.	-	-
5	199170	G	A	-	-	-	-	A	-	-	G/A	A	-	-
5	199192	T	C	-	-	-	-	C	-	-	T/C	C	-	-
5	199195	T	C	-	-	-	-	.	-	-	.	.	-	-
5	199229 *	A	G	-	-	-	-	G	-	-	A/G	G	-	-
5	199272 *	G	A	-	-	-	-	A	-	-	G/A	A	-	-
5	199274 *	A	G	-	-	-	-	G	-	-	A/G	G	-	-
5	199276 *	C	A	-	-	-	-	A	-	-	C/A	A	-	-
5	199317	G	A	-	-	-	-	.	-	-	.	.	-	-
5	199326		Insertion	hetero	hetero	hetero	hetero	.	hetero	hetero	.	.	hetero	-
5	199336 *	C	T	.	.	.	.	.	.	.	.	C/T	.	-
5	199353	T	Deletion	.	.	.	.	.	.	.	.	.	.	hetero
5	199409	T	C	.	C	T/C	T/C	C	T/C	T/C	T/C	C	.	T/C

The nucleotides differernt from the reference are indicated. Two nucleotides are shown for heterozygous sites. Dots indicate the same nucleotide sequences as in the reference. Genomic regions intervened with heterozygous indels were hardly decoded by the direct sequencing method and the undecoded nucleotides are indicated by hyphens. Insertions and deletions found are shown by "hetero" if the sites are heterozygous and by "homo" if the sites are homozygous. Note that only genetic variations found in the alignments of whole-genome shotgun sequences are shown and variations found only in this experiments are not shown for simplicity. Asterisks indicate positions, variations at which are represented by only one JP read.

Table S2. Numbers of genetic variations found in 11 different animals and in whole genome shotgun reads.

	Variations found in the validation experiment	Variations not found in the validation experiment
Variations found only in the JP dataset	67	23
Valiations found only in the US (US1 and US2) dataset	16	14
Valiations found in the JP and the US datasets	37	11
Variations not found in the whole genome shotgun reads	30	-

Table S3. Number of synonymous and non-synonymous/nonsense SNPs in genes encoding ribosomal proteins

Gene	Gene model	Number of synonymous SNPs	Number of Non-Synonymous/Nonsense SNPs
Genes encoding non-mitochondrial ribosomal proteins			
RPL3	KH.C2.198	5	0
RPL4	KH.C1.263 & KH.L18.139	8	0
RPL5	KH.C9.386	9	0
RPL6	KH.L41.49	10	0
RPL7	KH.C2.77	6	0
RPL7A	KH.C3.237	5	0
RPL8	KH.L123.1	6	0
RPL9	KH.C14.209	0	0
RPL10	KH.C12.107	2	0
RPL10A	KH.C2.28	5	0
RPL11	KH.L22.58	2	0
RPL12	KH.C9.73	2	1
RPL13	KH.S793.1	3	0
RPL13A	KH.C6.46	5	0
RPL15	KH.S595.6	12	0
RPL17	KH.C2.141	3	0
RPL18	KH.C9.355	3	2
RPL18A	KH.C8.109	1	0
RPL19	KH.C1.333	4	0
RPL21	KH.C2.744	1	0
RPL22	KH.C1.296	6	0
RPL23	KH.L24.2	2	0
RPL23A	KH.L152.7	3	0
RPL24	KH.C6.158	3	0
RPL26	KH.C7.70	0	0
RPL27	KH.L141.29	3	0
RPL27A	KH.C10.212	5	0
RPL28	KH.C9.87	3	0
RPL29	KH.S910.2	2	0
RPL30	KH.C2.631	1	0
RPL31	KH.C2.551	1	0
RPL32	KH.C5.166	1	0
RPL34	KH.S1840.1	1	0
RPL35	KH.C9.404	0	0
RPL35A	KH.C7.10	2	0
RPL36	KH.C7.196	0	0
RPL36A	KH.C14.360	0	0
RPL37	KH.C8.30	0	0
RPL37A	KH.C9.517	0	1
RPL38	KH.C8.20	0	0
RPL39	KH.L20.54	2	0
RPL40	KH.C4.189	1	0
RPL41	KH.C9.469	0	0
RPLP0	KH.C11.21	9	0
RPLP1	KH.C2.216	0	0
RPLP2	KH.C11.339	1	1
RPS2	KH.C5.184	6	0
RPS3	KH.C2.108	1	0
RPS3A	KH.C2.693	4	0
RPS4	KH.C12.188	7	0
RPS5	KH.C13.29	6	0
RPS6	KH.L40.23	5	1
RPS7	KH.S761.1	3	0
RPS8	KH.L133.9	1	1
RPS9	KH.C9.358	4	0

RPS10	KH.L170.19	3	0
RPS11	KH.C10.57	2	5
RPS12	KH.C7.264	6	0
RPS13	KH.C11.268	3	0
RPS14	KH.C14.231	2	0
RPS15	KH.S852.1	2	1
RPS15A	KH.L10.10	1	0
RPS16	KH.S406.5	0	0
RPS17	KH.C8.322	2	0
RPS18	KH.C12.47	4	0
RPS19	KH.C10.101	0	0
RPS20	KH.C8.308	2	0
RPS21	KH.L147.12	0	0
RPS23	KH.L59.2	1	0
RPS24	KH.C14.120	3	0
RPS25	KH.L95.1	1	0
RPS26	KH.C2.257	1	0
RPS27	KH.C3.248	1	0
RPS27A	KH.C10.239	6	0
RPS28	KH.C8.209	0	2
RPS29	KH.C1.115	0	0
RPS30	KH.C10.10	4	0
RPSA	KH.C11.148	6	0
Genes encoding mitochondrial ribosomal proteins			
MRPL1	KH.L40.19	3	0
MRPL2	KH.C8.860	0	0
MRPL3	KH.C2.227	2	0
MRPL4	KH.C7.55	6	5
MRPL9	KH.C1.843	8	0
MRPL11	KH.C3.38	2	0
MRPL13	KH.C1.86	4	1
MRPL14	KH.C7.386	7	1
MRPL15	KH.C2.450	18	4
MRPL16	KH.C8.756	7	0
MRPL17	KH.C12.593	2	3
MRPL18	KH.C3.723	7	5
MRPL19	KH.L153.19	4	2
MRPL20	KH.C4.529	12	0
MRPL21	KH.C14.487	5	3
MRPL22	KH.C1.975	6	2
MRPL23	KH.L9.5	3	0
MRPL24	KH.S164.8	2	0
MRPL27	KH.L110.2	2	0
MRPL30	KH.C2.510	6	4
MRPL33	KH.C7.236	2	0
MRPL35	KH.C1.1229	3	2
MRPL37	KH.C5.380	6	3
MRPL42	KH.C8.499	4	8
MRPL43	KH.C3.90	4	3
MRPL45	KH.S1090.1	9	7
MRPL47	KH.C7.228	7	1
MRPL48	KH.C8.294	2	5
MRPL49	KH.S425.13	9	7
MRPL52	KH.C1.328	2	0
MRPL55	KH.C8.32	0	0
MRPS2	KH.L96.74	5	1
MRPS5	KH.C3.121	8	1
MRPS6	KH.C1.1257	5	2
MRPS7	KH.C5.214	7	5
MRPS10	KH.C1.233	6	5
MRPS11	KH.C1.1274	1	3
MRPS12	KH.C9.151	0	1

MRPS14	KH.L55.1	1	4
MRPS15	KH.L153.71	6	1
MRPS16	KH.C9.179	2	0
MRPS17	KH.C4.354	3	1
MRPS18A	KH.C11.171	4	2
MRPS18B	KH.L102.6	4	2
MRPS22	KH.C1.209	3	9
MRPS23	KH.C12.420	8	2
MRPS24	KH.C12.465	5	1
MRPS25	KH.C1.220	3	2
MRPS30	KH.L96.36	11	5

Table S7. Primers used in the present study

Target chromosome	Forward primer	Reverse primer
Chromosome 1	5’-cattgttgcgtttgtgacg-3’	5’-atcgagacacgcagatgtgg-3’
Chromosome 2	5’-catcttctgtgattttgtgacg-3’	5’-cccaccagaatacattgtacg-3’
Chromosome 3	5’-atggttgcagaacggaagc-3’	5’-tcccaaccaggaaagatacg-3’
Chromosome 4	5’-gagactcgcaaagtggatgg-3’	5’-tttgccgctgtaactcttcc-3’
Chromosome 5	5’-accgagaacattgactgtgg-3’	5’-ctcatcatcgtgttgactgc-3’
